# Conjugative transfer frequencies of *mef*(A)-containing Tn*1207.3* to macrolide-susceptible *Streptococcus pyogenes* belonging to different *emm* types

**DOI:** 10.1111/lam.12213

**Published:** 2014-01-24

**Authors:** NF Hadjirin, EM Harrison, MA Holmes, GK Paterson

**Affiliations:** 1 University of West LondonLondon, UK; 2 Department of Veterinary Medicine, University of CambridgeCambridge, UK; 12213-curr-0001 Department of Veterinary Medicine, University of CambridgeMadingley Road, Cambridge, CB3 0ES, UK

**Keywords:** antibiotics, molecular epidemiology, streptococci, transposons

## Abstract

**Abstract:**

The aim of this study was to examine the gene transfer potential of *mef*(A)-containing Tn*120.3* to macrolide-susceptible *Streptococcus pyogenes* belonging to different *emm* types. Using the filter mating technique, Tn*1207.3* was transferred by conjugation to 23 macrolide-susceptible recipients representing 11 *emm* types. PCR analysis confirmed the presence of the *mef*(A) gene and the *comEC* junction regions of the Tn*1207.3* insertion in resultant transconjugants. Significant variation was found in the transfer frequency of Tn*1207*.*3* to different *Strep*. *pyogenes* strains, and this phenomenon may contribute to the differences in *mef*(A) frequency observed among clinical isolates.

**Significance and Impact of the Study**

The spread of antimicrobial resistance among pathogenic bacteria is an important problem, but the mechanisms of horizontal transfer between strains and species are often poorly understood. For instance, little is known on how macrolide resistance spreads between strains of the human pathogen *Strep. pyogenes* and why certain strains more commonly display resistance than others. Here, we show that *Strep*. *pyogenes* strains vary greatly in their ability to acquire a transposon encoding macrolide resistance by horizontal gene transfer *in vitro*. These data provide a novel insight into the transfer of antibiotic resistance between bacterial strains and offer an explanation for the differences in the frequency of resistance determinates and resistance seen among clinical isolates.

**Significance and Impact of the Study:**

The spread of antimicrobial resistance among pathogenic bacteria is an important problem, but the mechanisms of horizontal transfer between strains and species are often poorly understood. For instance, little is known on how macrolide resistance spreads between strains of the human pathogen *Strep. pyogenes* and why certain strains more commonly display resistance than others. Here, we show that *Strep*. *pyogenes* strains vary greatly in their ability to acquire a transposon encoding macrolide resistance by horizontal gene transfer *in vitro*. These data provide a novel insight into the transfer of antibiotic resistance between bacterial strains and offer an explanation for the differences in the frequency of resistance determinates and resistance seen among clinical isolates.

## Introduction

*Streptococcus pyogenes* (group A streptococci/GAS) remains an important human pathogen responsible for a wide variety of invasive and noninvasive infections including pharyngotonsillitis, skin and soft tissue infections and bacteraemia. Macrolides are widely used to treat GAS infections in patients allergic to *β*-lactam antibiotics. The development and spread of macrolide resistance among GAS throughout the world is therefore a major concern.

The drug efflux pump, encoded by the *mef* gene (currently represented by four subclasses A, E, I and O), is one of the most common mechanisms responsible for resistance to 14- and 15-membered macrolides in GAS and other species (Sutcliffe *et al*. 1996a; Varaldo *et al*. 2009). At least three mobile elements carrying the *mef*(A) gene have been identified in GAS: the Tn*1207.3* transposon (Santagati *et al*. 2003), the Φ*10394.4* phage chimera (Banks *et al*. 2003) and the Φ*m46.1* element (Brenciani *et al*. 2010). Studies indicate a significant prevalence of the *mef*(A) gene among GAS isolates in Europe and elsewhere. For instance, nationwide surveillance in Germany noted a frequency of 31% (Bley *et al*. 2011), while an Italian study found the prevalence of *mef*(A) among GAS to be 16% (Creti *et al*. 2005). Interestingly, there appears to be a skewed distribution of macrolide resistance genes among different GAS strains belonging to different *emm* types. The *mef*(A) gene is most frequently harboured by certain *emm* types such as *emm1*, *emm2*, *emm3*, *emm4*, *emm9*, *emm12* and *emm75*, whereas *emm* types such as *emm*22, *emm*77, *emm*87 and *emm*89 have not yet been documented to carry any *mef* determinant (Creti *et al*. 2005; Grivea *et al*. 2006; Silva-Costa *et al*. 2008; Wajima *et al*. 2013). These observations suggest differences in the capacity of GAS *emm* types to acquire *mef* genes, and while the horizontal gene transfer of *mef*(A)-bearing genetic elements has been demonstrated *in vitro* by conjugation (Giovanetti *et al*. 2003; Santagati *et al*. 2003) and by transduction (Di Luca *et al*. 2010), no comparison has yet been made of *mef* gene transfer frequencies among different GAS strains.

The aim of this study therefore was to examine the transfer frequency of *mef*(A)-containing Tn*1207*.*3* to diverse macrolide-susceptible recipients representing a variety of clinically important *emm* types.

## Results and discussion

Erythromycin-resistant transconjugants were detected in 21/23 of the tested recipient strains, with mean frequencies of transconjugants ranging from 7·20 × 10^−8^ to 1·13 × 10^−6^ conjugant/recipient (Table1). Statistical analysis showed that there was a significant difference in the conjugation frequency between strains (anova, *P* < 0·001). PCR confirmed that all 85 randomly selected transconjugants were positive for the *mef*(A) gene as well as the 5′ and 3′ junctions of Tn*1207.3-comEC*. All amplicons were of the expected size. Thus, growth on erythromycin-selective plates can be taken as being strongly indicative of *mef*(A) acquisition by the previously susceptible, *mef*A-negative recipient strains and the integration of Tn*1207.3* into the *comEC* locus.

**Table 1:** Strain characteristics and gene transfer frequencies of the *mef*(A)-bearing Tn*1207.3* element (SMH036) to macrolide-susceptible *Strep. pyogenes*

*emm* type	Recipient	Multilocus sequence type	Site of isolation	Mean frequencies of three transfers per recipient ± SEM
*emm*1	R366	ST28	Blood	9·83 × 10^−6 ^± 1·87 × 10^−6^
	R203	ST28	Blood	1·13 × 10^−6 ^± 6·46 × 10^−7^
	R057	ST28	Eye	3·86 × 10^−6 ^± 1·91 × 10^−6^
	R502	Not available	Blood	4·23 × 10^−6 ^± 1·85 × 10^−6^
*emm*2	R837	ST55	Blood	3·17 × 10^−6 ^± 3·52 × 10^−7^
	R007	ST55	Blood	2·37 × 10^−6 ^± 5·30 × 10^−7^
*emm*3	R032	ST315	Throat	1·20 × 10^−7 ^± 1·52 × 10^−8^
	R079	ST315	Throat	9·80 × 10^−7 ^± 1·72 × 10^−7^
*emm*4	R317	ST39	Blood	4·50 × 10^−7 ^± 4·93 × 10^−8^
	R211	ST39	Blood	6·47 × 10^−7 ^± 1·05 × 10^−7^
	R101	ST39	Ear	6·17 × 10^−6 ^± 3·75 × 10^−7^
*emm*9	R480	ST75	Blood	9·20 × 10^−7 ^± 1·89 × 10^−7^
	R097	ST75	Pus	1·60 × 10^−7 ^± 1·53 × 10^−8^
*emm*11	R098	ST22	Blood	8·13 × 10^−7 ^± 1·67 × 10^−7^
	R693	ST22	Blood	3·67 × 10^−7 ^± 1·86 × 10^−8^
*emm*12	R214	ST36	Blood	1·19 × 10^−7 ^± 5·01 × 10^−8^
	R113	ST36	Throat	3·97 × 10^−7 ^± 4·97 × 10^−8^
	R044	ST36	Throat	6·00 × 10^−7 ^± 8·88 × 10^−8^
*emm*59	R205	Not available	Blood	4·36 × 10^−8 ^± 8·60 × 10^−9^
*emm*77	R054	ST77	Skin	<3·77 × 10^−8^ (none detected)
	R115	ST77	Vagina	<1·08 × 10^−8^ (none detected)
*emm*81	R202	ST117	Blood	3·97 × 10^−7 ^± 1·48 × 10^−7^
*emm*87	R208	ST62	Blood	7·20 × 10^−8 ^± 8·99 × 10^−9^

The association of *emm* type with conjugative frequency is difficult to assess given the number of isolates of each *emm* type was low. However, the data show a trend for higher frequencies of conjugation among *emm*1 and *emm*4, seen in the region of 10^−6^ to 10^−7^ with lower rates seen particularly with the single isolates of *emm*59 and *emm*87, in the region of 10^−9^, Table1. These patterns correlate well with the observed frequency of *mef*(A) among clinical isolates belonging to these *emm* types, *mef*(A) being highly prevalent among *emm*1 and *emm*4 isolates but having not been reported among *emm*59 and *emm*87 isolates (Creti *et al*. 2005; Grivea *et al*. 2006; Silva-Costa *et al*. 2008; Wajima *et al*. 2013). These data also highlight the potential for the emergence of *mef*(A)-mediated resistance in previously susceptible *emm* types. Two isolates, both belonging to *emm*77, did not produce transconjugants above the lower limit of detection, Table1, suggesting that a low capacity for acquisition exists among *emm77*. This again correlates well with epidemiological data, and *emm77* has not previously been associated with *mef*(A). Interestingly, previous work using transduction also failed to transfer *mefA* into an *emm* 77 isolate (Di Luca *et al*. 2010). All seven *emm* types included here and reported previously to harbour the *mef*(A) gene were able to acquire it by conjugation *in vitro*. Taking only the *emm* types for which at least three isolates were included, *emm*1, *emm*4 and *emm*12, the higher mean frequencies of transconjugants seen with *emm*1 and *emm*4 isolates were statistically significant compared to those seen with *emm*12 isolates. This suggests that an association exists between *emm* type and the transfer frequency of Tn*1207.3* but the testing of a larger collection of isolates is needed to explore this further.

Together the results demonstrate that significant variability exists in *mef*(A) gene conjugative transfer rates between *Strep. pyogenes* strains and that this phenomenon may contribute, at least in part, to the different frequencies of *mef*(A) observed among clinical GAS strains. The mechanisms responsible for these differing rates of *mef*A transfer are not known, but M proteins have previously been proposed to act as barriers for horizontal gene exchange and thus may play a role (Schmitz *et al*. 2003). Furthermore, we demonstrate potential for the development of *mef*(A)-mediated erythromycin resistance in currently susceptible lineages.

## Materials and methods

### Bacterial isolates

Group A streptococci isolates were collected, as part of a separate study, from two London Hospitals, St. Mary’s and Paddington and Hammersmith, between 1993 and 2005 (McGregor and Spratt 2005). *mef*(A) recipient strains belonged to the following eleven *emm* types: *emm*1, *emm*2, *emm*3, *emm*4, *emm*9, *emm*11, *emm*12, *emm*59, *emm*77, *emm*81 and *emm*89. Strains were chosen based on their availability for study and because they include some of the most common lineages among clinical disease isolates in Europe (Steer *et al*. 2009) (Table1). They also include lineages with observed differences in their frequency of *mef*(A) prevalence (Creti *et al*. 2005; Grivea *et al*. 2006; Silva-Costa *et al*. 2008; Wajima *et al*. 2013). Isolate SMH036 (*emm*75) was used as the Tn*1207.3* donor and carries the Tn*1207.3* element inserted into the *comEC* locus. Discrimination of *mef* gene subclasses A, E, O and I by RFLP analysis confirmed the presence of only *mef*(A) in SMH036. SMH036 was confirmed to be resistant to erythromycin with a MIC of 8 mg l^−1^ and grew readily on 2 mg l^−1^ erythromycin-selective blood plates. It was, however, susceptible to fusidic acid and rifampicin. All recipient strains were confirmed to be susceptible to erythromycin (MIC < 0·015–0·12 g l^−1^) and unable to grow on selective blood agar plates containing 2 mg l^−1^ erythromycin used in screening for transconjugants. The wild-type recipients were also confirmed to be susceptible to rifampicin and fusidic acid, and resistant derivatives of these, used to differentiate transconjugants from SMH036, were generated by inducing to spontaneous mutations conferring resistance to rifampicin or fusidic acid.

### *In vitro* conjugative gene transfer of Tn*1207.3*

Filter mating was performed according to Giovanetti *et al*. (Giovanetti *et al*. 2002). Transconjugants were selected on blood agar plates containing either fusidic acid (25 mg l^−1^) or rifampicin (25 mg l^−1^) in addition to erythromycin (2 mg l^−1^). Transfers were performed in triplicate.

### Confirmation of *mef*(A) transfer

Putative transconjugants were tested for the presence of *mef*(A) by PCR using primers *mef*F/*mef*R as previously described (Sutcliffe *et al*. 1996b). Both Tn*1207*.*3*/*comEC* junctions were also examined by PCR, primers TnRjF/TnRjR (D’Ercole *et al*. 2005) spanning the 5′ junction and *comEC*F/*orf*LF (Santagati *et al*. 2003) spanning the 3′ junction. Donor SMH036 was used as a positive control for these PCRs. Transconjugants were randomly selected (3–6 per donor/recipient combination) for PCR validation with a total of *n *= 85. All recipient strains produced no amplicons in these PCRs.
